# Photocatalytic Degradation of Textile Dye on Blended Cellulose Acetate Membranes

**DOI:** 10.3390/polym14030636

**Published:** 2022-02-07

**Authors:** Abdullah M. Asiri, Valerio Pugliese, Francesco Petrosino, Sher Bahadar Khan, Khalid Ahmad Alamry, Soliman Y. Alfifi, Hadi M. Marwani, Maha M. Alotaibi, Debolina Mukherjee, Sudip Chakraborty

**Affiliations:** 1 Chemistry Department, Faculty of Science, King Abdulaziz University, Jeddah 21589, Saudi Arabia; sbkhan@kau.edu.sa (S.B.K.); kaalamri@kau.edu.sa (K.A.A.); salfaifi@kau.edu.sa (S.Y.A.); hmarwani@kau.edu.sa (H.M.M.); mmsalotaibi@kau.edu.sa (M.M.A.); 2 Department of Computer Engineering, Modeling, Electronics and Systems (D.I.M.E.S.), University of Calabria, Via-P. Bucci, Cubo-42A, 87036 Rende, Italy; valeriopugliese@libero.it (V.P.); f.petrosino@dimes.unical.it (F.P.); debolina.mukherjee@unical.it (D.M.)

**Keywords:** blended membrane, PES, textile dye, photocatalyst, water treatment, cellulose acetate

## Abstract

This work aimed to investigate the degradation performance of natural cellulose acetate (CA) membranes filled with ZnO nanostructures. Photocatalytic degradation of reactive toxic dye methylene blue (MB) was studied as a model reaction using UV light. A CA membrane was previously casted and fabricated through the phase inversion processes and laboratory-synthesized ZnO microparticles as filler. The prepared membrane was characterized for pore size, ultrafiltration (UF) performance, porosity, morphology using scanning electron micrographs (SEM), water contact angle and catalytic degradation of MB. The prepared membrane shows a significant amount of photocatalytic oxidation under UV. The photocatalytic results under UV-light radiation in CA filled with ZnO nanoparticles (CA/ZnO) demonstrated faster and more efficient MB degradation, resulting in more than 30% of initial concentration. The results also revealed how the CA/ZnO combination effectively improves the membrane’s photocatalytic activity toward methylene blue (MB), showing that the degradation process of dye solutions to UV light is chemically and physically stable and cost-effective. This photocatalytic activity toward MB of the cellulose acetate membranes has the potential to make these membranes serious competitors for removing textile dye and other pollutants from aqueous solutions. Hence, polymer–ZnO composite membranes were considered a valuable and attractive topic in membrane technology.

## 1. Introduction

Polymeric membranes significantly influence agro-food, water treatment, waste feed from the industrial stream, and textiles, including removing pollutants from freshwater. The membrane-based treatment provides better treatment and processes for concentrating the waste sources and reducing the active and reactive contaminants from industrial wastewater [1,2,3,4].

Membrane-based separation is extensively utilized to resolve critical energy and environmental challenges, and is widely used for water decontamination mainly because it can remove targeted particles or ions depending on membrane characteristics. One of the primary reasons for water contamination worldwide is rapid industrialization, and textile industries are one of the main contributors in creating this problem. On the other hand, sewage streams are hardly treated before being discharged into the environment. In the textile dying process, after dying the textile material, the residue dye (25–50%) goes as waste in aqueous solutions, which before discharge is infrequently treated. Several types of artificial dyes are being widely used in textile industries worldwide, potentially toxic to the environment. Most types of dye are not environmentally friendly because they have been classified as a poisonous colorant [5]. The dye employed in this study is methylene blue (MB), a basic dye used in textile industries, which is considered dangerous when it exceeds distinct concentrations due to its high toxicity. Sure enough, a high dosage of MB causes adverse effects on human health and marine pollution [6]. Moreover, most organic dyes are not degraded because they are highly resistant to environmental conditions, making removal from the waste stream a significant prerequisite [7] for many industries before discharging to the environment.

Therefore, it is essential to eliminate these textile dyes by adopting advanced, cost-effective, efficient technologies. Different conventional technologies are widely used to date for wastewater treatment. Among all the methods, photocatalytic degradation is an efficient process due to its low cost, high system efficiency, and easy operation processes [8,9]. Combining the membrane separation and inorganic photocatalysts is highly important due to several advantages and process costs. Not only that, but the ease of operations is also another aspect that needs to be addressed. Blending nanoparticles in the polymeric membrane matrix justify significant attention for many potential low-cost applications. Amongst the others, cellulose is the most abundant polymer, which is mainly used as supporting raw material for fiber technology. It is advantageous because it is biodegradable, environmentally friendly, biocompatible, and cost-effective. Cellulose acetate (CA) shows all the features listed above and represents one of the most used polymers for wastewater treatment membranes [10,11,12]. This is one of the first works that dealt with the innovative blended CA-ZnO membrane for textile dye treatment. Remarkably, the blend of polymer and nanoparticles successfully enhanced membrane hydrophilicity and photocatalytic capabilities.

Nanoparticles have emerged as solid options to standard materials, robust, high surface area heterogeneous photocatalysts, and catalyst support. In addition, combination of nanoparticles with polymeric membranes, is considered a potential and efficient technology for mineralizing pollutants into environmentally favorable compounds [13]. The nanoparticles possess a high-level surface-to-volume ratio, which enhances the visible surface area of the catalyst’s active component, improving the contact time between catalyst and reactants. Furthermore, a high level of surface-to-volume ratio (SVR) is required for a toxin particle to be adsorbed onto the surface of a photocatalyst for redox reactions (oxidation and reduction). In this way, the residence time increases, which helps for the complete degradation/mineralization of the contaminant [8,13].

Amongst different metal-derived oxides, zinc oxide (ZnO) is a promising due to its large variety of available nanostructures. They also have advantages over other catalysts since it has low cost, high surface reactivity, high and effective adsorption potential, and destructive sorbent capacity than other inorganic catalysts [14,15,16,17]. On the other hand, ZnO is also one of the studied metal oxides used frequently in membrane technology and membrane modifications for its capacity to inhibit the macro void formation on the membrane surface with improved mechanical strength [18,19]. Additionally, the use of ZnO nanoparticles significantly decreases membrane fouling and increases membrane permeation efficiency.

A few papers on the presented topic were previously published. Durthi et al. proposed a mixed matrix membrane of cellulose acetate blended with ZnO for arsenic removal from wastewater [20]. However, the solvent that could best suit the purpose of this work was N, N-dimethylformamide (DMF), a non-green, polar aprotic and a nucleophilic compound with multiple outstanding features and is well used both in membrane technology and in nucleophilic substitution reactions. Furthermore, being a polar solvent, it can stabilize the opposing transition states, promoting the removal of the dispatch group from its formamide part ([20] A).

Kusworo et al. proposed a hybrid cellulose acetate membrane for increasing eugenol content in clove oil [21]. Sheikh et al. treated different applications already existent for wastewater treatment with polymeric ZnO blended membranes [22]. Abu-Dalo et al. studied the photocatalytic degradation of methylene blue (MB) using polymeric membrane impregnated with ZnO nanostructures [23].

However, the presented works and many others available in the literature do not propose green solvents in membrane preparation. The choice of solvent is strictly related to the characteristics of cellulose acetate or the polymer used. Many are generally insoluble in water and many organic solvents due to the high polymerization degree and the significant chain compaction obtained through the multiple intermolecular hydrogen bonds generated by the hydroxyl groups inside the cellulose. The number of hydroxyl moieties substituted by acetyl groups is commonly known as the degree of substitution (DS).

Common solvents such as oxolane (THF), acetic acid methyl ester, dioxane, or acetone, can be used for DS higher than two. Since cellulose acetate is characterized by DS less than two, few solvents, including acetic acid or DMF/DMA, can dissolve this type of polymer. Since common organic solvents can cause severe environmental issues because they develop a flammable, volatile gas or waste which cannot be recovered and recycled, it should be essential to search for “green” cellulose extraction/dissolution methods. Green solvents, commonly referred to as ionic liquids (ILs), are organic salts with melting temperatures below 100 °C, which have attracted considerable appeal as eco-friendly options to traditional organic solvents. Thanks to their notable properties, such as high ionic conductivity, low flammability, high chemical/thermal stability, low vapor pressure, and ease of recyclability ([23] A,B).

An already published work by our research team proposed synthesis and characterization of composite cellulose acetate membranes [24]. The present work illustrates the advancement of polymeric blended membrane based on the CA/ZnO combination to reduce methylene blue from wastewater streams from textile and industrial processes. The innovation of the prepared CA-ZnO membrane is that it demonstrates increasing and great efficiency than other previously reported materials [25]. Combining ZnO nanoparticles with CA porous matrix decreases membrane fouling and increases permeation quality significantly. It shows how a CA membrane can represent an advantageous and cost-effective system in eliminating and degrading MB from polluted aqueous media. The separation capabilities of these membranes, besides their catalytic activity, permit a combined dye treatment process of photodegradation/filtration, which is another benefit.

## 2. Materials and Methods

### 2.1. Materials

Cellulose acetate (CA; average acetyl content of 39.7 wt%, average MW~50,000 (GPC) (Sigma Aldrich, Burlington, MA, USA) has been used as a polymer component for membrane preparation. The N, N-dimethylformamide (DMF) purity of 99% (Sigma Aldrich, Burlington, MA, USA) has been used as a solvent during polymer blending. Laboratory synthesized Zinc oxide (ZnO) has been employed as a membrane additive. A Glycerol (Honeywell, Morristown, NJ, USA) solution was also used in membrane washing operations. For the synthesis of nanostructured zinc nitrate hexahydrate Zn(NO_3_)_2_·6H_2_O (Lach-Ner, Tovární, Neratovice), urea >99% purity (NH2)2CO (Sigma Aldrich, Burlington, MA, USA), zinc acetate dihydrate >99% purity Zn(CH_3_COO)_2_·2H_2_O (Sigma Aldrich, Burlington, MA, USA), hexamethylenetetramine >99% purity (HMTA)C_6_H_12_N_4_ (Merck, New York, NY, USA), PEI, sodium citrate dihydrate >99% purity C_6_H_5_Na_3_O_7_·2H_2_O (Merck, New York, NY, USA) has been used. Methylene Blue was supplied by local manufacturers who use it for their day-to-day applications. The main components of UV source light were used from a mechanical solar simulator model no. 21117 (Newport, CA, USA).

### 2.2. ZnO Synthesis

In general, Zn(NO_3_)_2_ and HMTA equimolar solutions are used for ZnO nanostructure synthesis. The final composition of the solution was prepared by adding up extra chemicals depending on different experiments. An equimolar aqueous solution of 0.1 M hexamethylenetetramine and 0.1 M zinc nitrate were interspersed and stirred for 30 min, and at the end, both are mixed, stirred at 130 °C for 24 h. Finally, ZnO nanorods were grown using autoclave by hydrothermal synthesis method. The containers filled with samples were placed inside an oven with controlled temperature. The samples with the pre-dispersed ZnO layer were visibly dipped into the growth solution facing downward, then separated from the precipitate solution. Afterwards, the homogeneous solution was taken out from the oven and cooled at ambient temperature. Then, samples were removed and rinsed several times with distilled water and finally dried at 90–110 °C for 30 min to remove excess water.

### 2.3. Composite Membrane Preparation

The cellulose membranes were prepared by the phase inversion method [24,26]. The polymeric solution, obtained by dissolving the CA in DMF, has been stirred at 75 °C (using a water bath) for 3–4 h. Different amounts of ZnO were added into the polymeric solution and further optimized the amount of ZnO for the best membrane. Subsequently, the polymeric solutions were left at 60 °C for 8 h without stirring to remove air bubbles. After that, the polymeric solution was kept in the fume hood for around 12 h to remove other air bubbles in the vacuum environment. The mixture was stored in an ultrasonic bath at 60 °C for 8 h to prevent the solution from aggregating.

The degassed solution was poured onto a glass plate and then the membrane was cast using a casting knife (Elcometer 3450). The casted membranes were immediately immersed in a coagulation bath for one hour. The resulting flat sheet membranes exhibited a skin layer thickness of about 50 µm [27]. The precipitated membranes were removed from the coagulation bath and washed with distilled water to eliminate the excess amount of solvent.

The membrane washing needs to be executed several times to ensure all DMF solvent is completely removed from the membrane matrix. The wetting membranes were dried up in ambient conditions until a dry porous flat-sheet membrane was attained. Finally, the dried membrane was immersed in aqueous glycerol (10% *v*/*v*) in a water solution for preservation and future use. The composition of all suspensions is expressed by considering a weight percentage (wt%) of the ZnO concerning the total solute mass given in Table 1. An ultrasound bath operation was adopted to allow a very good dispersion of the ZnO particles as explained in previously published work.

### 2.4. Membrane Characterization

The physicochemical properties of the prepared membrane were investigated using scanning electron microscopy (SEM), X-ray diffraction (XRD), mechanical strength as well as FTIR, and thermal analysis (TA), including final degradation under UV conditions. A UV spectrophotometer performed UV-Vis Absorption Spectrum to characterize the synthesized ZnO.

SEM (Cambridge Zeiss LEO 400) microscope has been used to analyze the membrane surface morphology and cross-section. After being placed in liquid nitrogen, the membranes were cracked to keep unaltered the film structure and coated with gold to reduce the charging effects on the polymer surface.

Then, the membrane porosity was investigated by a capillary Flow Porometer CFP 1500 AEXL (Porous materials Inc. PMI, Ithaca, New York, NY, USA). A membrane portion has been wholly wetted with wetting liquid (Galwick, 15.9 dyne/cm). After that, tests were executed based on the wet up/dry up method. The bubble point measurement was performed calculating the pore size diameter, Dp, based on the reported Laplace’s equation (Equation (1)).

Dp = 4γcosθ/P(1)

where γ is the surface tension of the liquid, θ is the contact angle of liquid, and P is the external pressure.

Moreover, the membrane physical property characterization consisted of Tensile Strength and Young’s Modulus determination. A tensile testing machine (Zwick/Roell Proline Z005 equipped with a Load Cell Xforce P) was used. A crosshead speed of 3 mm/min was used during experiments, and the sample was 25 mm wide and 45 mm long.

The membrane surface hydrophilicity was investigated through water contact angle measurements using the tensile drop method at ambient temperature by a CAM 200 contact angle meter (KSV Instruments LTD, Helsinki, Finland). At least 4 measurements have been taken, depositing an ultrapure water drop (5 µL) on the membrane.

X-RD analysis with a Bruker equipment (D8 ADVANCE) with a monochromatic Cu K𝛼 radiation (λ = 0.154 nm) source operated at 40 mA and 40 kV between 20° and 70°, has been performed to confirm the existence of ZnO nanoparticles in the membrane. Finally, thermal analysis of the membranes has been performed using a simultaneous thermal analyzer (Netzsch, STA429 CD) under a nitrogen atmosphere at a heating rate of 10 °C/min. from 20 °C to 800 °C.

Pure water permeability tests investigated the permeation properties of the prepared membrane. A Millipore (XFUF07601) solvent-resistant Stirred Cell for 76 mm membranes was used. At least in triplicate, the experiments have been performed at three different transmembrane pressures (1.5, 2.5 and 3.5 bar). The temperature was the ambient one (25 °C). The permeate flux has been evaluated by using the reported equation:

(2) $$J=\frac{V}{A\times t}$$

where *J* is permeated flux (L/m^2^h), *V* is the volume of the accumulated permeate, *A* is the membrane surface area and the filtration time.

### 2.5. Catalytic Degradation of MB

**Figure 1:**
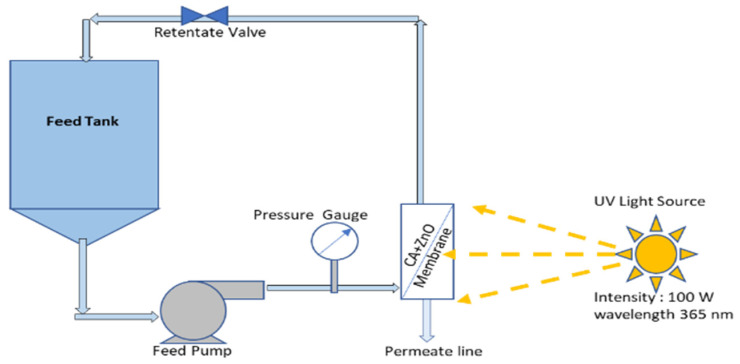
Photocatalytic membrane (CA-ZnO) reactor.

The photocatalytic performance of the prepared CA-ZnO membrane has been evaluated by measuring the degradation rate of the methylene blue (MB) under UV irradiation. The previously used Millipore membrane module was readapted as PMR to irradiate the membrane during UF experiment, as explained in Figure 1. As stated before, a mechanical solar simulator (model no. 21117, Newport, USA) was used to irradiate the membrane module. The permeate had a different structure regarding feed solution as they went through membrane pores and geometry. During the entire experimental period, samples were collected at a regular interval from the permeate stream and investigated into the spectrophotometer (see Section 2.6).

Our experimental work guarantees any possible adsorption of MB on the membrane pores present in the surface, and it has been achieved before the photocatalytic reaction. To achieve this, MB’s feed solution was continuously recirculated through the membrane module in the dark phase without UV. This process also allows the membrane module to stabilize the reaction parameters. Monitoring of permeate concentration was performed until we could see a steady state flux. After that, a solar simulator was switched on so that the UV could reach the membrane moreover the ZnO surface and activate it. This way, it triggered the catalytic reaction on the ZnO composite membrane pores.

The emission intensities on the reactor were kept at a constant value in the 315–400 nm range. During each new run, the degradation of MB was monitored over time.

### 2.6. Characterization of Permeate Product

UV–vis spectrophotometry at 664 nm was used to measure the MB concentration in the feed and permeate. To confirm the correctness of the measurements, quantitative analysis of the components present in the permeate solution was made using HPLC (Thermo Scientific Dionex UltiMate 3000 Photodiode Array Detector), which is also fitted with an C18 Reversed-phase LC column (Acclaim-120) working at 25 °C. The injection volume inside the column was 10 μL, whereas the eluent flow rate was 0.2 mL min^−1^. MB concentrations were a mixture of 34% water/34% acetonitrile/32% methanol in mobile phases. The permeate concentration in HPLC was measured from the peak as obtained in the chromatogram. Distinct peaks have been found attributed to the degradation products and non-degraded MB from the chromatographic graph. It is still under the investigation phase for concluding the result. The stability of the feed MB-UV absorbance spectrum ensured that no overlapping of this peak occurred with those due to degradation products from permeate phase.

## 3. Result and Discussion

### 3.1. UV-Vis Absorption Spectrum

**Figure 2:**
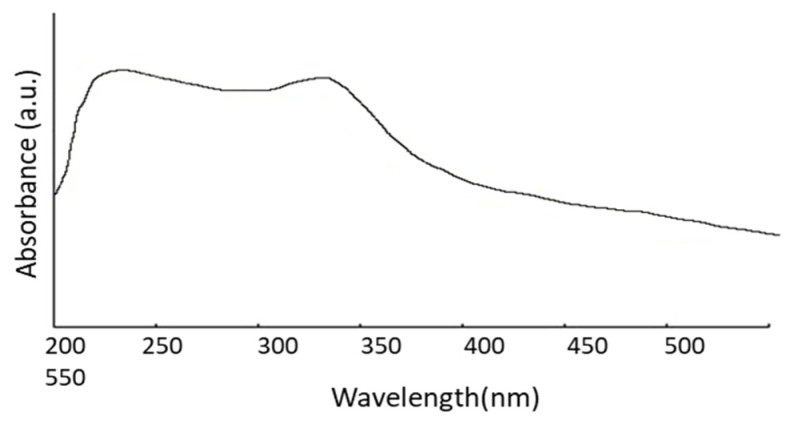
UV-Vis absorption spectrum.

The size of the nanoparticles plays an essential role in changing the entire properties of materials. Thus, the size evolution of semiconducting nanoparticles becomes necessary to explore the properties of the materials. UV-visible absorption spectroscopy is a widely used procedure to investigate the optical properties of nanoparticles. The absorption of synthesized ZnO nanopowder is illustrated in Figure 2. It exhibits a strong absorption band at about 355 nm. An excitonic absorption peak is found at about 258 nm due to the ZnO nanoparticles, which are situated much below the bandgap of 358 nm. It is also evident that the significant sharp absorption of ZnO indicates the monodispersed nature of the nanoparticle distribution.

**Figure 3:**
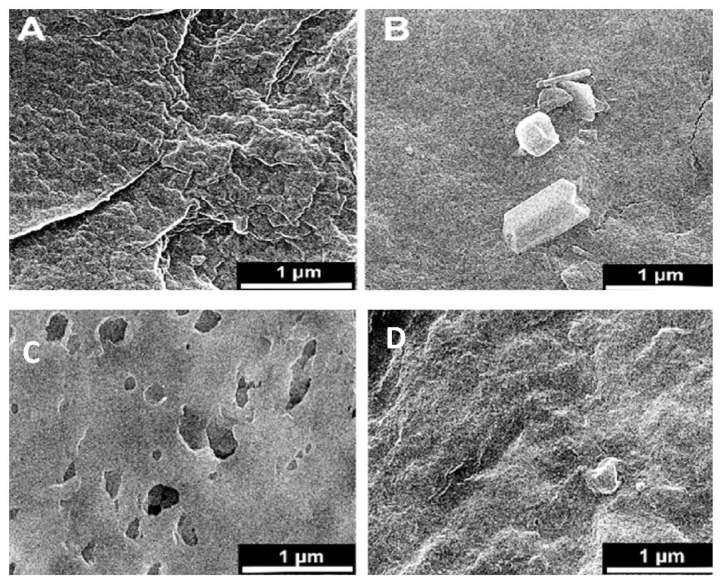

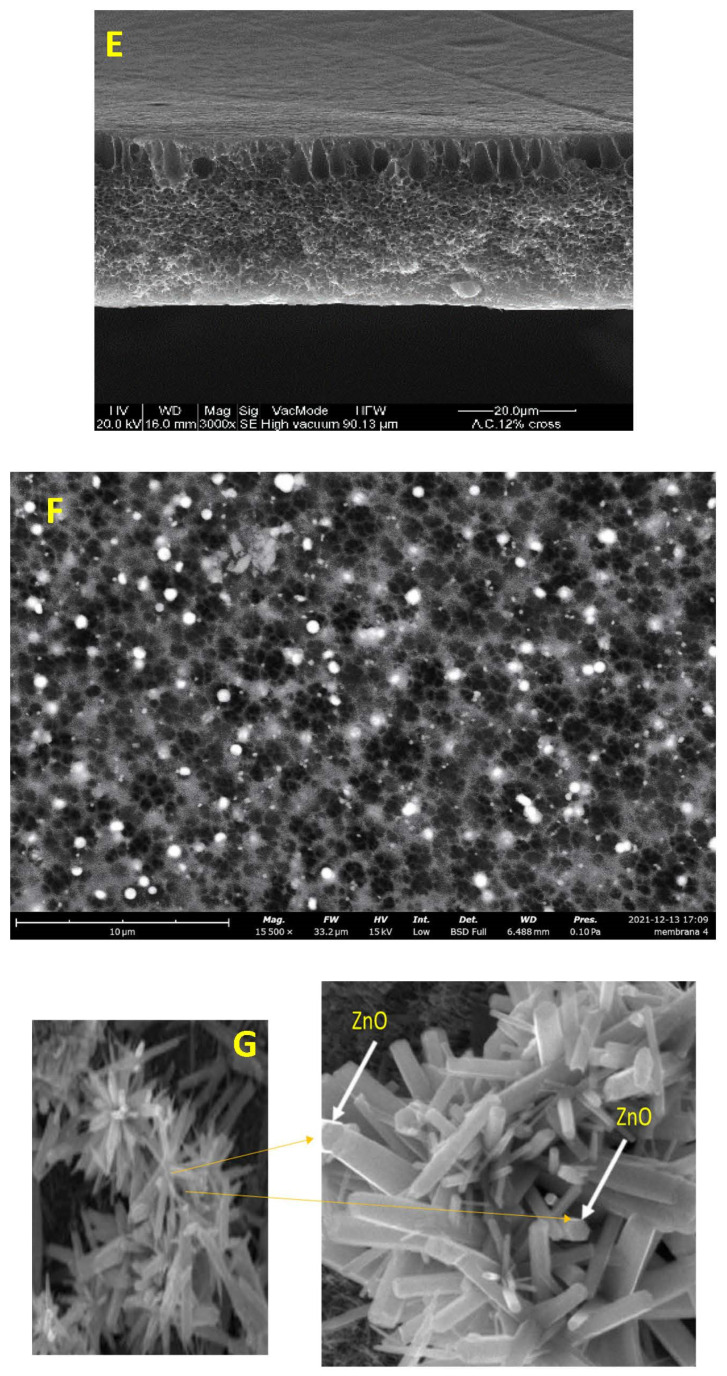
Surface SEM images of an uncoated membrane (**A**–**C**), 1% ZnO blended membrane (**B**–**D**), cross-section of the asymmetric CA membrane 12% CA + ZnO (**E**), top view of the asymmetric CA membrane 12% CA + ZnO, (**F**) inside view of the asymmetric CA membrane 12% CA + ZnO an individual ZnO flower (**G**).

The average particle size in a nano colloid can be calculated from the absorption onset from UV-vis absorption spectra by using an effective mass model given in Equation (3). The bandgap 𝐸∗ can be approximated by [28].

(3) $$E^{*}=E_{g}^{bulk}+\frac{h^{2}\Pi^{2}}{2er^{2}}\left(\frac{1}{m_{e}^{*}m_{0}}+\frac{1}{m_{h}^{*}m_{0}}\right)-\frac{1.8e}{4\pi \in \in_{o}r}-\frac{0.124e^{3}}{h^{2}{\left(4\pi \in \in_{o}\right)}^{2}}{\left(\frac{1}{m_{e}^{*}m_{0}}+\frac{1}{m_{h}^{*}m_{0}}\right)}^{-1}$$

where $E_{g}^{bulk}$ is the bulk band gap expressed in eV, *h* is Plank’s constant, *r* is the particle radius, *m_e_* is the electron effective mass, *m_h_* is the hole effective mass, *m*_0_ is free electron mass, *e* is the charge on the electron, *ε* is the relative permittivity, and *ε*_0_ is the permittivity of free space. ZnO has small effective gatherings (*m_e_* = 0.26, *m_h_* = 0.59), which is why band gap enlargement is expected in case the size of the particle is less than 4 nm. To find the ZnO particle, the following mass model equation (Equation (4)) is used [29].

(4) $$r\left(nm\right)=\frac{-0.3049+\sqrt{-26.23012+\frac{10240.72}{\lambda_{p}}\;\left(nm\right)}}{-6.3829+\frac{2483.2}{\lambda_{p}}\;\left(nm\right)}$$

where *λ_p_* is peak absorbance wavelength in nm. ZnO nanoparticles show an absorbance peak at about 258 nm, corresponding to the particle size of 2.07 nm. The SEM pictures below have also confirmed this result (Figure 3E,F).

### 3.2. Membrane Characterization: Microscopic Analysis of Membrane Surfaces

Microscopic SEM analyses have evaluated the effect of CA polymer concentration on the membrane morphology. The prepared membrane exhibited an asymmetric cross-section structure, with a thin selective layer and finger-like and sponge-like pore structures in the sub-layer (see Figure 2). The formation of the skin layer is for the instantaneous demixing of solvent and non-solvent during the phase inversion process [30]. This structure is due to the high mutual diffusivities of water and DMF during the phase separation [31]. In addition, the formation of macro-voids is obtained when the diffusion rate of the non-solvent into the polymer-poor phase overcomes the solvent diffusion rate. An increase in the polymer concentration determined a decrease in the dense skin layer. This result is attributed to the higher viscosity of the dope solution that blocks the diffusion exchange between solvent (DMF) and non-solvent (water). In addition, unlike a membrane containing only CA, on a blended one, the presence of the ZnO determined an increase in the suspension viscosity [19] and, as a consequence, the diffusion of the non-solvent decreased. Thus, the formation of macro-voids is suppressed, and so a higher number of pores is obtained in the skin layer (see Figure 3A–D).

However, adding the ZnO increased the pore amount (Figure 3C,D) because their addition strongly influences the thermodynamic and kinetic factors during the membrane formation prepared by the phase inversion technique [19]. The addition of the inorganic nanoparticles caused an increase in suspension viscosity. Thus, the diffusion of the non-solvent decreased, so the formation of macro-voids is suppressed, and a higher number of pores is formed in the skin layer. As you can also see, the presence of ZnO particles homogeneously distributed throughout the membrane (Figure 3E,F). Figure 3G also indicates flower-like ZnO nanostructure growth during the ZnO synthesis. The SEM micrograph 3G reveals some ZnO rods with hexagonal structures in the top layer.

### 3.3. Membrane Characterization: Mechanical Property

**Figure 4:**
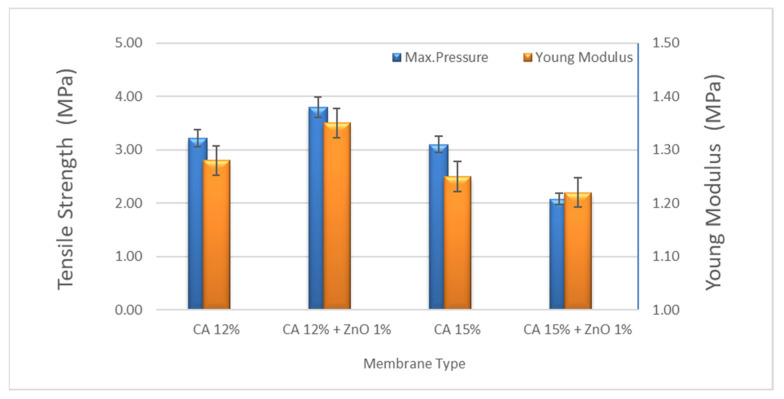
Mechanical strength of CA and CA + ZnO nanoparticles (diff wt%).

The tensile strength and the Young’s modulus of the best-prepared membrane resulted in 3.21 and 1.28 MPa for CA12 and 3.80 and 1.35 MPa for CA12-Z1, respectively. As reported before, the mechanical stability of the composite membranes increased with the ZnO concentration up to 1 wt% of ZnO. Hence, in conjunction with the increasing ZnO content greater than 1 wt%, a progressive decrease in mechanical stability due to the formation of nanoparticle clusters was observed, with a consequent weakening of the material resistance [32]. According to the existing literature, a ZnO concentration of 1 wt% was chosen to optimize the membrane mechanical properties. Consequently, as part of this work, only the photocatalytic activity of the composite membrane filled with 1 wt% ZnO nanoparticles was evaluated (Figure 4). The mechanical properties of the 15% CA membrane are reduced by adding ZnO particles because the polymer network is too dense, and a reverse effect with ZnO particles added was observed. On the other hand, adding ZnO in the polymer matrix makes the polymer solution unstable due to the incorporation of inorganic components and the formation of clusters. The higher the ZnO we added, the less intense the membrane became.

### 3.4. Membrane Characterization: Contact Angle Measurement

The water contact angle measurements of the CA membrane and the membrane blended with ZnO. The nanoparticles blended membrane shows enhanced hydrophilicity, as reported elsewhere [33]. In particular, ZnO nanoparticles embedded in the membrane surface can form hydrogen bonds with water molecules, resulting in the increased adsorption capacity of water and improved hydrophilicity of the membrane [34,35]. The contact angle values are reported in Table 2. In the case of CA15, the membrane was not permeable as no flux was detected. This is because the higher CA clusters make a very dense membrane, making it utterly impermeable until 5 bar of pressure.

### 3.5. Membrane Characterization: XRD Analysis

**Figure 5:**
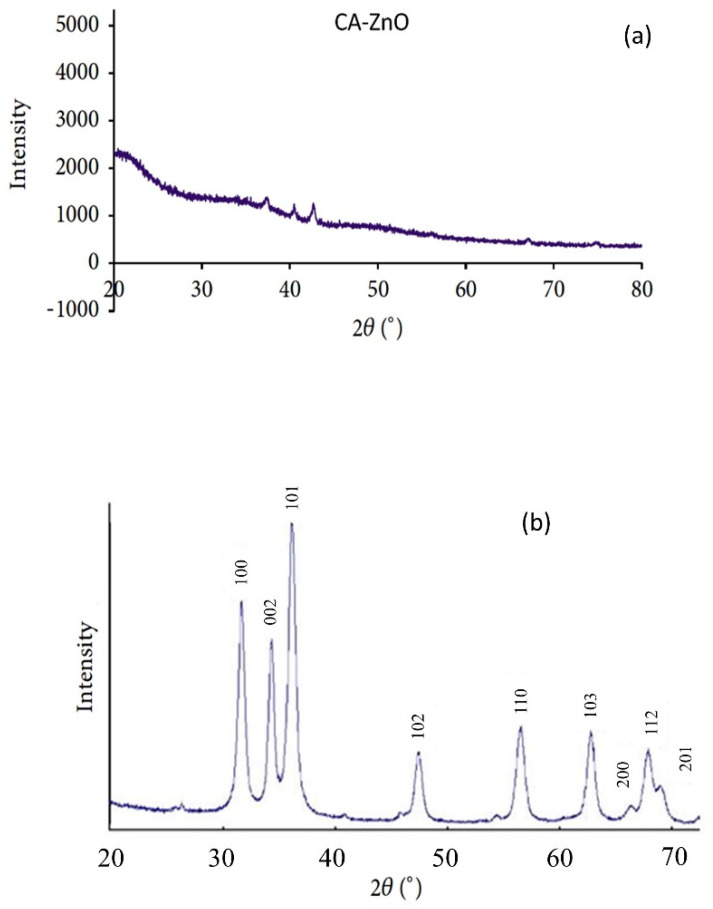
XRD analysis of (**a**) CA- ZnO membrane and (**b**) ZnO nanoparticles.

The existence of ZnO nanoparticles in the membrane polymer matrix has been verified by XRD analysis. Figure 5a,b show the CA-supported ZnO nanoparticle membrane’s diffractograms (Figure 5a) and the unsupported ZnO nanoparticle. The unsupported ZnO nanoparticles exhibited dominant peaks at 2*θ* angles of 36.91°, 39.98°, 42.17°, and 55.78°, which were described to the (100), (002), (101), (102), and (110) planes of the ZnO crystal structure form, respectively. This corresponds to the prominent characteristic peaks of zinc oxide nanoparticles. On the other hand, the CA-supported composite CA-ZnO membrane showed the distinct diffraction peaks of the ZnO nanoparticles and neat membrane, as expected for such a composite. As we can see, the ZnO/CA composite membrane had peaked at 2*θ* angles at of 35.62°, 37.73°, 30.20°, 53.25°, and 42.17°. These diffraction peaks proved the presence of crystalline ZnO nanoparticles. Similar results have been observed with ZnO incorporated in other polymer matrices, corresponding to the unsupported ZnO nanoparticles. The XRD results were an indication that ZnO nanoparticles were present in the prepared ZnO/CA composites membranes.

### 3.6. Membrane Characterization: Thermal Analysis

**Figure 6:**
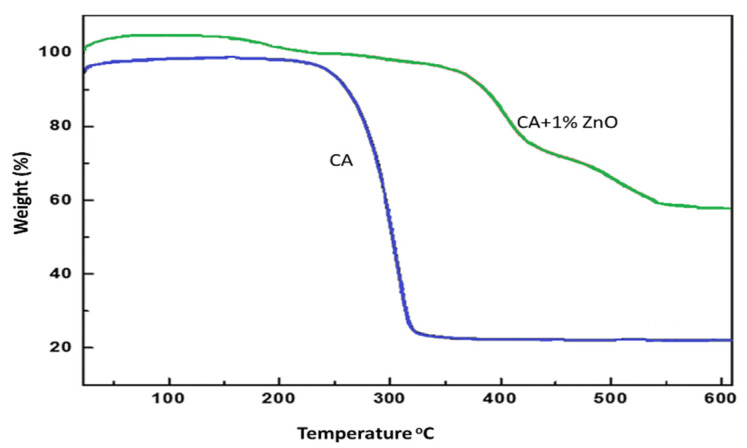
TGA results increase decomposition temperature for (0.0 and 1.0 wt%) of ZnO on CA membranes.

The thermal analysis (TGA) provides information on the decomposition temperature (DTemp). It is defined as the temperature at 6% weight loss. A simultaneous thermal analyzer (Netzsch, STA429 CD) was used to investigate the weight loss of both the bare CA and composite ZnO membranes. The decomposition temperature of the pure CA and CA/ZnO membrane followed a similar trend. The temperature gradually increased with the increasing addition of ZnO on CA membranes (Figure 6). The addition of ZnO nanoparticles improved the thermal stability of the CA membranes. In ultrafiltration experiments, the effect of the polymer concentration and ZnO addition on the permeability have been considered. The permeability of the CA membranes decreased with increased polymer content in the dope solution, owing to the reduction in the macro-void formation [30]. In addition, the membranes prepared at a CA concentration of 15 wt% did not permeate in the investigated range of transmembrane pressure. All the casted membranes were used to find the best permeable membrane. The test was replicated several times to minimize the experimental errors, which is around 5% in our case. The blended membranes increased the permeability explained by considering the coupled action of improved hydrophilicity and the increased membrane porosity.

### 3.7. Membrane Characterization: Permeability Test

The water permeability of the prepared membrane was equal to 457 L/(m^2^h/bar). The obtained value entirely agrees with the literature [36,37,38,39]. The blended membranes increased permeability by considering the combined action of improved hydrophilicity and the increased membrane porosity [40,41].

### 3.8. Membrane Characterization: Photodegradation Studies

The ZnO-blended CA membrane contains both organic (polymer) and inorganic (ZnO) components. The photocatalytic degradation for ZnO photocatalyst was investigated [42,43,44]. The CA-ZnO membrane evaluated the photocatalytic degradation of MB, a well-known organic dye performed with a stable solution at room temperature [45,46].

**Figure 7:**
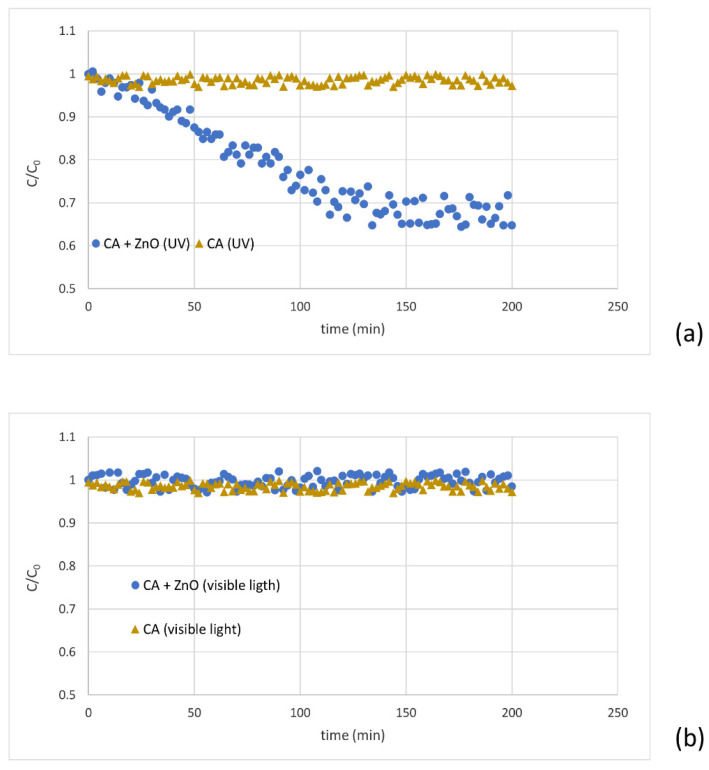
MB’s degradation under UV light (**a**) and visible light (**b**) for a virgin and blended membrane.

The degradation of MB in the presence of the CA-ZnO membrane under the UV treatment was measured at room temperature under UV lamp irradiation. UV was mainly radiated at 365 nm with the power of 100 W, 230 V, and 50 Hz frequency for 200 min, leading to the degradation of the dye (MB reduction of more than 30%) (Figure 7a). This result confirmed that ZnO is effective to decompose the dye in water. The photocatalytic mechanism of the composite membrane loaded with the ZnO during UV-light irradiation, as reported by Huang et al., the photo-generated electron-hole pairs react with O_2_ and H_2_O absorbed on the surface of ZnO nanoparticles with the formation of O_2_− and OH−, which contributes to the MB degradation [47]. The UV light’s electrons generated within ZnO will fill the valence band holes or alter with the adsorbed oxygen on the ZnO surface. Eventually, the MB molecules are degraded by the reactions with the radicals; simultaneously, oxygen ions are generated in contact with adsorbed water molecules. Consequently, hydroxyl groups and hydroxyl OH− radicals originated.

Moreover, different runs were performed with the same membranes irradiated by visible light. After almost 120 min of feed recirculation beneath solar light, the concentration of MB in the permeate phase achieved a steady state. However, for the virgin membranes, the permeate concentration remained unchanged (under UV and without UV), confirming that the composite membrane has a regulating factor for the degradation of MB (Figure 7b).

## 4. Conclusions

In this work, ultrafiltration ZnO modified cellulose acetate membrane (CA/ZnO) were prepared by phase inversion method. The photocatalytic degradation of the methylene blue was investigated with a newly fabricated membrane. It has been demonstrated that the composite membrane can degrade the toxic MB with less harmful components in laboratory conditions. Different characterization has been performed with the composite membrane, confirming nanocatalyst homogeneous distributions on the polymeric membrane matrix. The membrane with 12% CA with 1% ZnO showed higher degradation efficiencies in respect to the other membrane.

Several other process control parameters are under investigation, demonstrating that the CA membrane blended with ZnO can work for the MB or other toxic chemical degradation. The said technology has some limitations as well, due to improper system configuration. Developing an industrially viable large-scale system needs further design improvement and process optimization. Although we successfully demonstrated the concept, it needs further development to optimize different fluid dynamics and process parameters. Grafting and blending of ZnO is another challenge that needs to be identified and to maximize membrane separation performance and selectivity towards different feeds. It is worth mentioning that, to perform long-term stability of the system under various conditions and parameters, efficient degradation of targeted pollutants and their simultaneous separation must be safely ensured. All these areas are the subjects of further process development and future studies.

## Figures and Tables

**Table 1:** Membrane composition.

Sample	Cellulose (%)	ZnO (%)	DMF (%)	Cellulose (g)	DMF (g)	ZnO (g)
CA12	12	0	88	2.4	17.6	0
CA12-Z1	12	1	87	2.4	17.4	0.2
CA15	15	0	85	3	17	0
CA15-Z1	15	1	84	3	16.8	0.2

**Table 2:** Water contact angle of the prepared membranes.

Membrane Code	Permeability (LMH/bar)	Contact Angle
CA12	220	82.2 ± 0.7
CA12-Z1	445	77.1 ± 2.3
CA15	0	82.7 ± 2.1
CA15-Z1	0	76.0 ± 2.3

## Data Availability

The data that support the findings of this study are available from the corresponding author upon valid reasonable request and pending authorization after patent grant.
